# A dense linkage map for a large repetitive genome: discovery of the sex-determining region in hybridizing fire-bellied toads (*Bombina bombina* and *Bombina variegata*)

**DOI:** 10.1093/g3journal/jkab286

**Published:** 2021-08-17

**Authors:** Beate Nürnberger, Stuart J E Baird, Dagmar Čížková, Anna Bryjová, Austin B Mudd, Mark L Blaxter, Jacek M Szymura

**Affiliations:** 1 Research Facility Studenec, Institute of Vertebrate Biology, Czech Academy of Sciences, 603 65 Brno, Czech Republic; 2 Department of Molecular and Cell Biology, University of California, Berkeley, Berkeley, 94720 CA, USA; 3 Tree of Life Programme, Wellcome Sanger Institute, Hinxton, Cambridge CB10 1SA, UK; 4 Department of Comparative Anatomy, Jagiellonian University, 30-387 Kraków, Poland

**Keywords:** hybrid zone, targeted capture, sex-determining region, segregation distortion, anurans, linkage map, large-scale synteny, population pileups diplotyping, genome assembly

## Abstract

Genomic analysis of hybrid zones offers unique insights into emerging reproductive isolation and the dynamics of introgression. Because hybrid genomes consist of blocks inherited from one or the other parental taxon, linkage information is essential. In most cases, the spectrum of local ancestry tracts can be efficiently uncovered from dense linkage maps. Here, we report the development of such a map for the hybridizing toads, *Bombina bombina* and *Bombina variegata* (Anura: Bombinatoridae). Faced with the challenge of a large (7–10 Gb), repetitive genome, we set out to identify a large number of Mendelian markers in the nonrepetitive portion of the genome that report *B. bombina vs B. variegata* ancestry with appropriately quantified statistical support. Bait sequences for targeted enrichment were selected from a draft genome assembly, after filtering highly repetitive sequences. We developed a novel approach to infer the most likely diplotype per sample and locus from the raw read mapping data, which is robust to over-merging and obviates arbitrary filtering thresholds. Validation of the resulting map with 4755 markers underscored the large-scale synteny between *Bombina* and *Xenopus tropicalis.* By assessing the sex of late-stage F2 tadpoles from histological sections, we identified the sex-determining region in the *Bombina* genome to 7 cM on LG5, which is homologous to *X. tropicalis* chromosome 5, and inferred male heterogamety. Interestingly, chromosome 5 has been repeatedly recruited as a sex chromosome in anurans with XY sex determination.

## Introduction

Past hybridization leaves a clear signal in present-day genomes when unbroken, introgressed chromosome segments from another taxon are discovered. Such local ancestry tracts are irrefutable evidence of hybridization because they cannot be explained by either convergence or ancestral polymorphism (Rieseberg *et al.* 2000). Present-day hybrid zones set the stage for ongoing and future introgression. Against the backdrop of emerging reproductive isolation, some ancestry blocks may traverse the zone of contact and spread into the opposite gene pool either by neutral diffusion or at a rate proportional to their selective advantage (Barton 1979). As recombination breaks up two differentiated genomes into smaller segments (Barton 1983), local ancestry tracts are the natural units of inheritance in a hybrid zone (Baird 2006). For a given distribution of their lengths, likely combinations of hybrid zone age and selection regime may be inferred (Baird 1995). Local, transient distortions in the length distribution pinpoint genomic regions under strong selection (Sedghifar *et al.* 2016). Recent advances in genomic library technology (Meier *et al.* 2021) and population genomic theory (*e.g.*, Uecker *et al.* 2015; Hvala *et al.* 2018; Janzen *et al.* 2018; Sachdeva and Barton 2018; Shchur *et al.* 2020) harness the rich information contained in local ancestry tracts. They promise a step change in our understanding of selection and recombination in hybrid zones and of the process of speciation (*e.g.*, Powell *et al.* 2020; Meier *et al.* 2021).

The hybridizing fire-bellied toads *Bombina bombina* and *Bombina variegata* provide a textbook example (Urry *et al.* 2020) of abundant hybridization in typically narrow contact zones, despite ancient divergence (MRCA 3.2 mya, Nürnberger *et al.* 2016) and profound ecological differentiation (Szymura and Barton 1991; Szymura 1993; Yanchukov *et al.* 2006). Yet, insight into the mosaic of ancestry tracts within individuals and across the hybrid zone has so far eluded us because genomic resources for the large (7–10 Gb, Gregory 2021) and highly repetitive *Bombina* genome have been lacking. Inference from whole-genome resequencing of hundreds of samples is currently neither an option, nor in any case necessary, to uncover the haplotype structure. That goal is well served by a dense linkage map, which we present here based on three-generation experimental crosses between *B. bombina* and *B. variegata.*

We set out to identify a large number of Mendelian markers in the nonrepetitive fraction of the genome that report *B. bombina vs B. variegata* ancestry with appropriately quantified statistical support. We opted for targeted enrichment (reviewed in Jones and Good 2016) based on a new draft assembly of a *B. variegata* genome reported here and published *Bombina* transcriptomes (Nürnberger *et al.* 2016). This reduced representation approach (Davey *et al.* 2011) allowed us to filter out repetitive regions before selecting enrichment targets, obviated the need to infer exon-intron boundaries (as in exome capture, Neves *et al.* 2013) and, compared to methods based on restriction enzyme digestion, promised greater reproducibility and more even target coverage for this large genome (Jones and Good 2016). *Bombina* belong to the superfamily Discoglossoidea, which split ∼200 Mya from other anuran lineages with available genome assemblies (Feng *et al.* 2017). Capture probes derived from *Xenopus* or *Hyla* are thus not expected to work well in *Bombina* (Hedtke *et al.* 2013; Hutter *et al.* 2019). Enrichment success across taxon boundaries declines sharply in the range of 5–10% absolute sequence divergence, *d*_xy_ (Hedtke *et al.* 2013; Jones and Good 2016; Hutter *et al.* 2019). The distribution of *d*_xy_ between *B. bombina* and *B. variegata* has a mean of 0.0202 and a mode at 0.013 (Nürnberger *et al.* 2016). We, therefore, expected reliable cross-taxon enrichment for the great majority of targets as well as an abundant supply of ancestry-informative markers.

This approach produces read data centered on target intervals that are each only a few hundred base pairs long. Given our *a priori* knowledge of genome size and map length (2600 cM, Morescalchi 1965), recombination within target intervals should be exceedingly rare. We, therefore, treat each interval as a nonrecombining locus for which we wish to infer genotypes, based on *B. bombina* and *B. variegata* ancestry. Whenever more than one variant position exists in a given interval, an aggregate measure of ancestry is needed. For enrichment data, this can be complicated for two reasons. First, read coverage of a given interval is typically highest in the center and drops off sharply at either end (Chevalier *et al.* 2014; Harvey *et al.* 2016). Variant positions can thus vary greatly in read support. Second, mis-mapped reads from other parts of the genome can generate spurious variation signal, such as consistently heterozygous sites. Different genotypes may thus be called for different positions of the same interval and relative read support is a poor indicator of marker reliability. Preliminary variant calling analyses that we carried out highlighted these complications. We know of no rigorous, automated method to infer the best supported interval-spanning genotype, or diplotype, from multiple called genotypes at individual sequence positions. We, therefore, developed an approach that extracts the total ancestry information across all sequence positions of an interval. For each locus and sample, we computed the likelihoods of the three possible diplotypes in an F2 cross and used this information at the map making stage. In using all of the data without arbitrary filtering thresholds, by-passing the genotype call stage, and in propagating diplotype uncertainty, we followed the recommendations of Nielsen *et al.* (2011). Analyses of fourfold degenerate sites in *Bombina* transcriptomes (Nürnberger *et al.* 2016) indicated that between-taxon divergence exceeds within-taxon diversity by at least a factor of ten. Our three-diplotype inference scheme should thus capture the bulk of genetic variation segregating in our crosses.

Local ancestry inference is sensitive to errors in the linkage map. In a hybrid zone, long tracts are associated with recent immigration from the periphery. In addition, tracts with variants that reduce hybrid fitness persist for relatively shorter times and are therefore longer on average (Sedghifar *et al.* 2016). Mistyped and mis-mapped loci bias the tract length distribution by inflating the pool of short tracts and fragmenting true long ones (Gravel 2012). We, therefore, carried out additional analyses to assess the errors associated with the diplotyping method and the linkage map. In particular, we investigated the large-scale synteny between *Bombina* and *Xenopus tropicalis*. Since Anurans are a documented example of karyotypic conservatism or chromosomal bradytely (Bush *et al.* 1977; Baker and Bickham 1980; Marks 1983), we expected low chromosome variation across ∼200 million years of anuran evolution (Feng *et al.* 2017).

As a first application of the new linkage map, we identified the sex determining (SD) region in *Bombina*, which together with the majority of amphibians lack heteromorphic sex chromosomes (Eggert 2004; Ma and Veltsos 2021). Diplotype estimates were coupled with histological estimates of F2 progeny sex. While our three-diplotype inference scheme precludes estimation of separate male *vs* female recombination rates and the direct identification of a sex-linked haplotype, sex-biased segregation of these diplotypes in the F2 generation nonetheless pinpoints the SD region. This approach was applied to recent linkage maps in *Aedes aegypti* (Fontaine *et al.* 2017) and *X. tropicalis* (Mitros *et al.* 2019) and is explained below. In a further extension of our approach, we also inferred the SD mechanism.

In amphibians, the expected degeneration of the Y (or W) chromosome (Charlesworth and Charlesworth 2000) may be counteracted either by frequent turnover of sex chromosomes (Miura 2017; Jeffries *et al.* 2018) and/or very rare X-Y (or Z-W) recombination events (*e.g.*, in sex-reversed adults, Perrin 2009; Stöck *et al.* 2011; Guerrero *et al.* 2012; Rodrigues *et al.* 2018). Evidence for sex chromosome turnover and/or changes in heterogamety comes not just from comparisons among closely related species but also from ongoing transitions within species. For example, a distinct sex chromosome karyotype exists in each of three Swedish populations of *Rana temporaria* (Rodrigues *et al.* 2016; Toups *et al.* 2019). Separate W, Z, and Y chromosomes segregate in natural populations of *X. tropicalis* (Furman *et al.* 2020). Moreover, two species of *Bufo* toads with different SD systems hybridize in nature (*B. bufo*: ZW, *B. spinosus*: XY, Dufresnes *et al.* 2020). Intriguingly, hybridization may trigger such transitions. Detailed studies of the Japanese wrinkled frog, *Glandirana rugosa*, suggest that initial hybridization of two distinct XY populations triggered the establishment of three new SD systems (2× ZW and 1× XY), most likely in response to biased sex ratios in the hybrids (Miura 2007, 2017; Ogata *et al.* 2018).

Given the strong selection on and rapid divergence of SD systems (Coyne and Orr 2004), the map location of the *Bombina* SD region will be important for our analyses. In some hybrid zones, sex-linked as opposed to autosomal loci have formed steeper clines suggestive of stronger gene flow barriers (*Oryctogalus*, Carneiro *et al.* 2013; *Gryllus*, Maroja *et al.* 2015; *Hyla*, Dufresnes *et al.* 2016). On the other hand, striking cases of sex-linked introgression have been found and attributed to genetic conflict over the sex ratio (*Mus*, Macholán *et al.* 2008; *Drosophila*, Meiklejohn *et al.* 2018). Knowledge of the location of the SD region in *Bombina* will thus be critical for the analysis of their hybrid zones.

## Materials and methods

### Laboratory crosses

A male *B. v. variegata* from Obidowa (near Nowy Targ, Poland; sample acc. # ERS3926742) was crossed with a female *B. bombina* from Wodzisław Małopolski (Poland; sample acc. # ERS3926743) in 2014. Eighty F1 offspring were raised to maturity, and one F1 male was crossed with two F1 females to produce two F2 families (families 6 and 7 in the following; see Supplementary File S1 for husbandry, offspring rearing, and F1 sample accessions). The F2 offspring were raised to advanced metamorphosis (Gosner stage 42–44, Gosner 1960) and were humanely killed by MS222 (Ethyl 3-aminobenzoate methanesulfonate) overdose. For 80 offspring of family 6 and 82 offspring of family 7, the gonads with mesonephroi were dissected and fixed in Bouin’s solution (Kiernan 1990), while the remaining tissue was frozen. Toe clips were collected from the *B. bombina* grandmother and each of the F1 offspring under MS222 anesthesia. The *B. variegata* grandfather was euthanized by MS222 overdose and dissected for whole-genome sequencing. Tissue samples for DNA extraction were kept at −80°C.

### Whole-genome sequencing

DNA was extracted from muscle tissue of the *B. variegata* grandfather using the Invisorb Spin Tissue Minikit (Stratec, Germany). PCR-free TruSeq libraries with mean insert sizes of 350 bp (*n* = 8) and 550 bp (*n* = 2) were prepared by Edinburgh Genomics and sequenced on the Illumina HiSeq X, producing 6.67 × 10^9^ (350 bp insert) and 1.05 × 10^9^ (550 bp insert) read pairs (150 bp, PE). Adapter removal and quality trimming were carried out with bbduk (BBMap suite v.36.76, B. Bushnell, sourceforge.net/projects/bbmap/). Parameters for adapter removal were *k* = 23, mink = 8, and edist = 1 for R1 and *k* = 23, mink = 8, and edist = 2 for R2. Quality trimming parameters were trimq = 20, maq = 25, and minlength = 50. Genome size was estimated from unassembled reads with the *preqc* module of the String Graph Assembler (SGA, v. 0.10.15) (Simpson and Durbin 2012; Simpson 2014) using a subset of 1.1 × 10^9^ read pairs. All libraries were evenly represented in this and subsequent analyses.

### Genome assemblies

**Figure 1:**
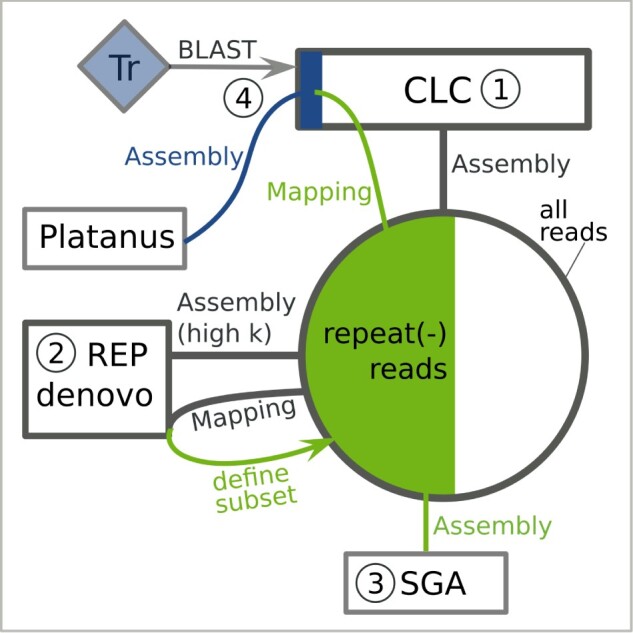
Overview of *B. variegata* genome assemblies. The large circle represents the total read set. Assemblies are numbered 1–4. hi K, highly over-represented kmers (at least 100x average frequency); Tr, *B. v. variegata* transcriptome; repeat(−) reads, repeat-subtracted portion of the total read set. CLC contigs with a BLAST+ hit (query: transcriptome) are represented by a blue rectangle.

A subset of 1.29 × 10^9^ read pairs (approximately 45× genome coverage) were assembled with the CLC Genomics Workbench (v. 9.5.3) (Qiagen, Hilden, Germany) using default parameters (Figure 1, step 1). Repeat sequences were assembled with REPdenovo (v. 2017-02-23) (Chu *et al.* 2016) with default parameters except MIN_REPEAT_FREQ = 100 (Chong Chu, *pers. comm*.; Figure 1, step 2). REPdenovo produced an unmerged version of all assembled repeats and a merged version by combining repeats with more than 90% identity. All quality-trimmed reads were mapped to the unmerged REPdenovo output with Bowtie2 (v. 2.2.3) (Langmead and Salzberg 2012), and the 52% of read pairs that did not map were extracted as the repeat-subtracted read set (Figure 1, green section). We queried the merged REPdenovo output against Repbase (Jurka *et al.* 2005; Bao *et al.* 2015) with the Censor tool (Kohany *et al.* 2006, blastn and tblastx, vertebrate database, last accessed July 31, 2020). Following Rogers *et al.* (2018), we annotated each merged REPdenovo contig with the highest scoring match and mapped a subset of 7.43 × 10^7^ read pairs (approximately 2.64× genome coverage) to the merged REPdenovo output with Bowtie2 (v. 2.2.3) (Langmead and Salzberg 2012). Mean mapped read coverage per contig was divided by 2.64 (*i.e.*, the approximate genome coverage) to estimate copy number.

The repeat-subtracted read set was assembled with SGA and Platanus, and sequences identical in these new assemblies and the previous CLC assembly were considered for bait design. For the SGA (v. 0.10.15) (Simpson and Durbin 2012) assembly, we followed the steps in the example assembly of a human genome (see the ./src/examples/ directory of the SGA distribution) using a subset of 1.12 × 10^9^ read pairs (approximately 40× genome coverage; Figure 1, step 3). For the Platanus (v. 1.2.4) (Kajitani *et al.* 2014) assembly, we first extracted CLC contigs that matched the published *B. v. variegata* transcriptome (Nürnberger *et al.* 2016) and 125 gene sequences from public databases with a minimum sequence identity of 90% with BLAST+ (v. 2.2.3) (Camacho *et al.* 2009). The transcriptome assembly consisted of 73,079 transcripts *[*∼ Trinity (v. 2014-04-13p1) components, Grabherr *et al.* 2011], and for 24,540 of these, open reading frames had been predicted. Extracted CLC contigs are represented by a blue rectangle in Figure 1 (step 4). Reads that mapped to these contigs with Bowtie2 (v. 2.2.3) (Langmead and Salzberg 2012) were assembled with the Platanus (v. 1.2.4) (Kajitani *et al.* 2014) assemble step.

### Candidate sequences and bait design

Candidate sequences for bait design were selected from the CLC assembly based on uniqueness, correct assembly, and minimal redundancy. We considered subsets of CLC contigs to be unique if they did not have any matches to other CLC contigs, based on an 85% sequence identity threshold with BLAST+ (v. 2.2.3) (Camacho *et al.* 2009). CLC contig sequences with exact matches (minimum length 100 bp) in the SGA and Platanus assemblies were deemed correctly assembled. Coverage and variant information (“bubbles”) provided by Platanus was used to flag overmerged sequences (see Supplementary File S1 for details). To minimize the proximity of enrichment targets (local redundancy), the CLC assembly was scaffolded against the *B. v. variegata* transcriptome assembly (Nürnberger *et al.* 2016) using SCUBAT2 (G. Koutsovoulos, https://github.com/GDKO/SCUBAT2, commit b03e770). For each SCUBAT2 path (*i.e.*, a set of contigs linked by exons from a single transcript), we identified the longest sequence section that was unique, correct, and lacked excessive variation. We also selected candidate sequences in CLC contigs (minimum length 5 kb) that were not included in any SCUBAT2 paths. These were filtered as previously described, except that exact matches were not confirmed against the Platanus assembly. Finally, all candidate sequence positions with a BLAST+ (v. 2.2.3) (Camacho *et al.* 2009) alignment against the unmerged REPdenovo output were hard masked.

We submitted 6400 candidate sequences (minimum length 500 bp; 4400 with known gene association) to Arbor Biosciences (Ann Arbor, Michigan, USA) for bait design and synthesis. For each of 5000 enrichment targets, four 100 base baits were designed that aligned with 50 base offsets to a 250 base sequence stretch (2x tiling). Baits were designed according to the strictest in-house criteria (no BLAST+ match to the CLC assembly with *T_m_* > 60°C, no “N” positions, %GC between 25 and 55, no RepeatMasker matches, and ΔG > −8).

### Enriched genomic libraries and sequencing

Genomic DNA was extracted from the F0 *B. bombina* grandmother, the three F1 parents, and the 162 F2 offspring using the Invisorb Spin Tissue Minikit (Stratec, Germany). DNA concentrations were measured by Qubit fluorometer (Invitrogen, USA) and normalized to 50 ng/µl. DNA extractions were then fragmented with the Bioruptor Pico (Diagenode, Belgium) using 7 cycles of 30 s fragmentation and 60 s cooling, which resulted in a mean fragment length of approximately 250 bp. Libraries were constructed from the fragmented DNA using the KAPA HyperPrep Kit (Kapa Biosystems, South Africa) per the manufacturer’s instructions, except all reaction volumes were halved. Dual indexed TruSeq-like adapters were added by ligation of “universal stubs,” followed by 8 cycles of PCR using indexed primers, as described by Glenn *et al.* (2019). SpriSelect beads (Beckman Coulter, USA) were used to size select the libraries, eliminating high molecular weight fragments with a 0.6× bead to sample volume ratio and low molecular weight fragments with a 1× ratio. Libraries were pooled in equimolar ratios (number of samples: 1, 2, or 4) and concentrated to 7 µl with 1× SpriSelect beads. The library pools were enriched using the myBaits target capture kit (Arbor Biosciences, Ann Arbor, Michigan, USA) with the custom baits. Hybridization was run at 65°C for 20 h. Enriched libraries were amplified with universal P5 and P7 primers during 11 cycles of PCR (PCR conditions as per the KAPA HyperPrep Kit). Amplified libraries were purified using 1× SpriSelect beads and mixed in equimolar ratios.

We tested the enrichment success and the effect of pooling libraries (1, 2, or 4 per enrichment reaction, including mixtures of the two taxa) using a single run of the Illumina MiSeq (v2 flow cell, 150 bp, PE). Because there was no apparent detriment to enriching four libraries in one reaction, this level of pooling was used for the entire dataset, excluding four instances with fewer than four samples. Enriched libraries of the *B. bombina* grandmother, the three F1 parents, and all 162 F2 offspring were sequenced on one lane of the Illumina NovaSeq (S1 flow cell, 150 bp, PE) by Edinburgh Genomics. An enriched library of the *B. variegata* grandfather was included in the Miseq test.

### Mapping reference

Because the enriched libraries span beyond the 250 bp bait regions, we used the “Assembly by Reduced Complexity” (ARC) package (v. 1.1.4-beta) (Hunter *et al.* 2015) to determine the mapping reference for each target. ARC bins read pairs based on the bait region to which they map and computes a unique *de novo* assembly for each bin with SPAdes (v. 3.9.0) (Nurk *et al.* 2013). This process is iterative, with the last *de novo* assembly used as the reference for the next mapping round until contig lengths stop increasing. From the enriched read-set of the F0 *B. variegata* adult, assemblies were obtained for 4850 targets. These were aligned against the CLC target contigs using BLAST+ (v. 2.2.3) (Camacho *et al.* 2009) in order to eliminate any sequence erroneously added to assembly termini and to resolve chimeric assemblies (McCartney‐Melstad *et al.* 2016). This screen resulted in mapping references for 4763 targets (see Supplementary File S1 for details). For the remaining 237 targets, the entire CLC contig was used as the reference. We constructed an analogous mapping reference for the *B. bombina* grandmother.

### Read mapping and diplotyping

**Figure 2:**
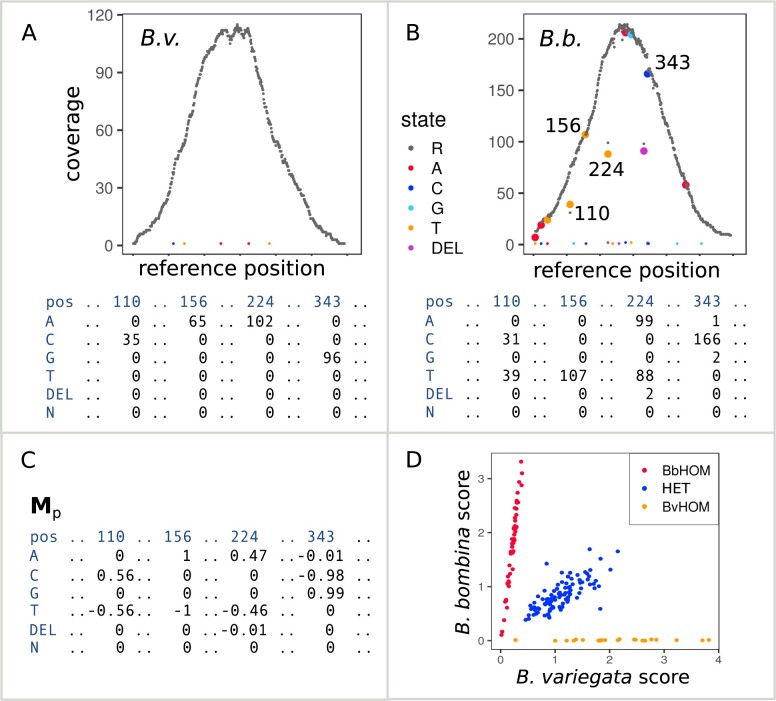
Polarization of the raw read coverage. The top two plots show the raw read coverage along the reference sequence of locus 332172 for F0 *B. variegata* (A) and F0 *B. bombina* (B). The *B. variegata* individual is homozygous for the reference state (R) at all sequence positions, whereas the *B. bombina* individual has a number of variants. Two homozygous (156 and 343) and two heterozygous (110 and 224) variant positions are highlighted. For these four, the matrix entries are listed below the plots. A polarized matrix, ***M***_p_, is computed from these read counts (see text, C), in which sequence states associated with *B. variegata* have positive entries and sequence states associated with *B. bombina* have negative entries. For each sample, raw read counts are then multiplied by ***M***_p_. Average positive entries and average negative entries result in a *B. bombina* score and a *B. variegata* score, respectively, and when plotted in a coordinate system (D), samples can be assigned to three clusters representing BbHOM, HET, and BvHOM. Note that the heterozygous variants (B) do not interfere with the clustering into three diplotypes.

The enriched sequence data were processed as previously described to produce repeat-subtracted reads sets. These were mapped with Bowtie2 (v. 2.2.3) (Langmead and Salzberg 2012) to the *B. variegata* mapping reference and, for a few samples, to the *B. bombina* analog to estimate mapping bias. Duplicates were flagged with Picard (v. 2.6.0) (Broad Institute 2019) MarkDuplicates and indels were realigned with GATK v. 3.7 (McKenna *et al.* 2010). For each bait interval, the mapped read data were summarized using Samtools (v. 1.4) (Li *et al.* 2009) mpileup and PoPoolation2 (Kofler *et al.* 2011) mpileup2sync. Command lines for these analyses are listed in Supplementary File S1. The resulting summary files contain, for each sample and locus, a matrix of *n* columns (*n* = number of reference positions) and six rows (sequence states of A, C, G, T, N, and DEL) of the counts of reads supporting each sequence state at each position. Note that insertions cannot be represented in this matrix of *reference* coverage. Figure 2 displays these data in compact form for one locus and for the F0 individuals: *B. variegata* (A) and *B. bombina* (B). Across all reference positions, the coverage of the reference state (R, *B. variegata*) is plotted in gray, whereas the coverage of variant states is shown in color. The matrix entries for four of the variant positions are displayed in panels A and B. The summary matrices were analyzed using a “Fast Vector” (FastVec) Mathematica (v. 12.0) (Wolfram Research, Inc. 2019) script. For algorithm details see Supplementary File S1. The aim is to define the best-supported haplotypes, one for each taxon, such that the combined likelihood of diplotypes (*B. variegata* homozygote, BvHOM; heterozygote, HET or *B. bombina* homozygote, BbHOM) inferred for each sample is maximized. FastVec part (1) employs a heuristic to generate candidate haplotypes. These are passed on to part (2) which coestimates the maximum likelihood (ML) taxon haplotypes and the ML diplotypes of all samples under a Mendelian model. Part (1) avoids the computational load of per-reference-position-state estimation combinatorics and instead applies a clustering approach to all samples’ read data for a given locus. First, for the two F0 grandparents, the counts are divided by the column totals to obtain frequencies. Subtracting the resulting *B. bombina* frequency matrix from the *B. variegata* frequency matrix gives a polarized matrix where positive entries represent sequence states that are more common in *B. variegata*, and negative entries are states more common in *B. bombina*. Signed entries are then weighted with respect to the support for this distinction in each matrix column (at each position). For a given position *i*, we compute the significance *Sig*(*i*) of the likelihood ratio test on the raw read counts of the two grandparents, comparing the hypotheses they were drawn either from the same or from different multinomial distribution(s). All matrix elements in column *i* are then multiplied by [1—*Sig(i*)]. This gives the initial weighted polarized matrix, ***M***_p_ (Figure 2C). The raw read count matrix for each sample is multiplied by ***M***_p_. The means of the positive and negative entries express the average weighted read coverage of sequence states associated with the *B. variegata* grandfather and the *B. bombina* grandmother, respectively, for that sample. When these positive and negative scores are plotted in a coordinate system, samples at a given locus fall into (typically) three clusters representing the three diplotypes (BbHOM, HET, and BvHOM; Figure 2D), with low coverage (and/or low power) individuals’ data near the origin.

***M***_p_, so far estimated from two individuals, is then updated using the combined raw read count matrices of the two clusters closest to the axes, *i.e.*, those containing the “purest” *B. bombina* and *B. variegata* individuals in the coordinate system, respectively. After this high-coverage ***M***_p_ update, separation of clusters such as Figure 2D was unchanged or improved. Note that more than three clusters could form if more than two haplotypes were segregating. We reduced the combined read counts of each of these clusters to a strict majority consensus, giving us a set of candidate haplotypes for the Mendelian likelihood analysis in part (2).

While the clusters near the axes were unlikely to be truly homozygous (due to for example sequencing error and overmerging), we could still determine the one haplotype pair across all possible pairs that best explained (with ML) all samples’ data: each individual’s raw read matrix must be explicable as a homozygous or heterozygous combination of this haplotype pair. ML determination requires computation of the likelihood of every individual’s matrix for every candidate haplotype pair and each diplotype state. These likelihoods are sufficient to coidentify the ML taxon haplotype pair, its support relative to other pairings, the ML diplotype for each individual and the support of those calls. These computations are robust to error, contamination (homozygote clusters: deviation from the 0° and 90°, respectively), and enrichment bias (heterozygote cluster: deviation from 45°). In a final step, we summed the combined data for all individuals identified as BvHOM to refine the estimate of the *B. variegata* haplotype, and similarly for the *B. bombina* haplotype. Supplementary File S1 gives a full description of the outputs of all these coestimates and estimates for each locus. Supplementary File S2 provides the Mathematica script.

We re-scored the 327 (6.5% of the total) loci that did not show the expected diploptypes in the F0 (BvHOM and BbHOM) and F1 (HET, HET, and HET) individuals. For each locus, coverage plots as in Figure 2 were produced for the five F0 and F1 samples. High-coverage variants that segregated in the F1 generation were selected by hand and annotated in a variant list extracted from the raw read matrices. A custom script then used these annotated variants to rescore all samples for each of the 327 loci.

### Linkage map

The linkage map was constructed with Lep-MAP3 (v. 0.2) (Rastas 2017), after recoding the diplotypes BbHOM, HET, and BvHOM as genotypes AA, AC, and CC in the Lep-MAP3 input file. The most likely diplotype was coded as 1, and the support estimate was provided for the other two diplotypes. We specified the three-generation pedigree in the input file in order to obtain a joint map across both F2 families (Supplementary File S3). We followed the Lep-MAP3 pipeline (lowest path in Figure 1 of Rastas 2017) with recommended parameters (https://sourceforge.net/p/lep-map3/wiki/LM3%20Home/#introduction), except halfSibs = 1, noSexFiltering = 1, grandparentPhase = 1, and LodLimit = 19 (see Supplementary File S1 for command lines). The most likely sex-averaged locus order in each linkage group (LG) was determined from 40 (LG1 and LG2) or 20 (all other LGs) replicate runs of the OrderMarkers2 module using the Kosambi mapping function. Segregation distortion (χ^2^ estimates) per locus and family were calculated with Lep-MAP3 (Filtering2 module). We applied the following significance thresholds to the χ^2^ data: (1) a Bonferroni correction, dividing α  =  0.05 by the number of chromosome arms (24) in *Bombina* (Morescalchi 1965; Manilo *et al.* 2006), as recommended by Fishman and McIntosh (2019) and (2) the Benjamini and Hochberg (1995) false discovery rate.

To test the robustness of the linkage map to outlier loci and to the frequent assignment of multiple loci to the same map position, we prepared a reduced dataset with only one locus per map position. First, all loci with higher than expected segregation distortion were removed. For a given bin (map position with ≥ 2 loci), we counted for each locus pair the differences in inferred diplotypes across all 162 F2 individuals. Loci associated with pairwise differences larger than three were removed and one of the remaining loci of that bin was picked at random (see Supplementary File S1 for details). Lep-MAP3 was then rerun with the reduced data set and map positions were compared between the full and the reduced map. For the reduced map, pairwise LOD scores and recombination fractions per LG were computed and plotted with OneMap v. 2.1.3 (Margarido *et al.* 2007) in R v. 4.0.3 (R Core Team 2020).

### Histology

F2 gonads with mesonephroi, fixed in Bouin’s solution, were dehydrated in an ethanol series, embedded in paraplast (Sigma), and sectioned. The 8 μm sections were stained with hematoxylin and picroaniline according to Debreuill’s trichrome procedure (Kiernan 1990). Images were taken with a Nikon Eclipse E600 light microscope. Sex of individuals was assessed from gonad morphology (Piprek *et al.* 2010; Piprek 2013, see Supplementary Figure S1). For some of the 162 samples, all ethanol accidentally evaporated just prior to embedding. This resulted in poor quality sections that made sex determination uncertain (*n *=* *34) or impossible (*n *=* *7).

### Finding the SD region

We estimated an SD bias that arises due to the nature of the crosses. In the F1s, the SD haplotypes of the heterogametic parent are taxon-labeled. That is, given the direction of the F0 cross (male *B. variegata* × female *B. bombina*) and assuming an XY system, the F1 male passes the *B. variegata*-labeled Y haplotype to his sons and the *B. bombina*-labeled X haplotype to his daughters. At the SD locus, we therefore expected F2 males to be only BvHOM or HET and F2 females to be only BbHOM or HET, both in equal proportions (Supplementary Figure S2). The same pattern would be expected in a ZW system. We quantified this sex-homozygote bias with the following equation, where *N* [] is a count:

$$b=\frac{N\left[\mathrm{maleBbHOM}\right]+N\left[\mathrm{femaleBvHOM}\right]}{N\left[\mathrm{HOM}\right]}.$$

With an equal sex ratio and no heterozygote deficit, the null expectation is *b *=* *0.5. At the SD locus itself, *b* should be zero.

In order to identify the heterogametic sex, we needed to define a sex-limited haplotype. If this haplotype is sufficiently distinct, more than three diplotype clusters will form in the *B. bombina-B. variegata* coordinate system, with strongly sex-biased clusters. For each locus, we ranked clusters by their proportion of males, *p*_m_, and identified, in descending order, the minimal set of clusters that jointly contained more than 50% of all males. We termed the average *p*_m_ of these clusters *pMaleInMaleClusters.* At an autosomal locus, the proportion of males in each cluster will be around 0.5, and *pMaleInMaleClusters* must therefore be about 0.5*.* At the extreme, there may be a cluster that contains the majority of all males and no females, such that *pMaleInMaleClusters =* 1*.* Note that the sex-homozygote bias in the three-cluster case (BbHOM, HET, and BvHOM; see above) produces less extreme estimates. At the SD locus, the BvHOM cluster would be entirely male (*p*_m_ = 1) and contain 50% of all males. The HET cluster (expected *p*_m_ = 0.5) would need to be added to obtain more than 50% of all males, such that *pMaleInMaleClusters* would be 0.75. We similarly computed *pFemaleInFemaleClusters*.

## Results

### Genome characteristics and assemblies

**Figure 3:**
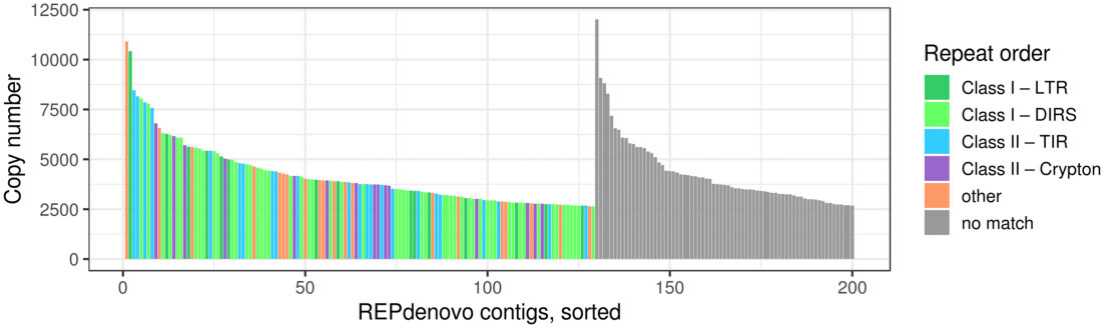
The distribution of repeat types. We show the 200 REPdenovo contigs with the highest copy number. Transposable element orders represented by more than 10 contigs in this set are identified by color. The classification follows Wicker *et al.* (2007). Contigs without a match in Repbase (blastn and tblastx) are labeled as “no match” and ordered separately. LTR, long terminal repeat retrotransposon; DIRS, *Dictyostelium* intermediate repeat sequence; TIR, terminal inverted repeat DNA transposon.

From kmer frequencies [SGA (v. 0.10.15) (Simpson and Durbin 2012; Simpson 2014) *preqc*], we obtained a *B. variegata* genome size estimate of 7.61 Gb. A second estimate of 8.12 Gb based on the same dataset and computed with GenomeScope 2.0 (Ranallo-Benavidez *et al.* 2020) was provided by K.S. Jaron (pers. comm.). The average of these two, 7.87 Gb, is used throughout this study. The GenomeScope analysis also confirmed an earlier report (Olmo *et al.* 1982) that *Bombina* is diploid. We explored the repeat content assembled by REPdenovo (v. 2017-02-23) (Chu *et al.* 2016) and extrapolated the repeats’ presence in the *B. variegata* genome based on the calculated copy number. The merged REPdenovo output contained 6039 contigs, totaling 4.5 Mb, with 3689 contigs matching known Repbase repeats (Jurka *et al.* 2005; Bao *et al.* 2015). The most common repeats were DIRS retrotransposons (Poulter and Goodwin 2005), which were identified in 1539 REPdenovo contigs and featured prominently in the set of 200 contigs with the highest copy number (Figure 3). The estimated total copy number of DIRS contigs was 807,858, covering 0.75 Gb of the *B. variegata* genome, or just under 10% of the total genome of 7.87 Gb. DNA transposon superfamilies that accounted for significant portions of the *B. variegata* genome included Crypton (0.21 Gb), hAT (0.19 Gb), and Mariner (0.10 Gb; see Supplementary Table S1 for a full list). The 2350 REPdenovo contigs that did not have any Repbase matches were estimated to cover 0.52 Gb of the *B. variegata* genome and include the REPdenovo contig with the highest copy number (Figure 3).

We assembled the *B. variegata* F0 grandfather’s genome using the CLC Genomics Workbench (v. 9.5.3) (Qiagen, Hilden, Germany), SGA (v. 0.10.15) (Simpson and Durbin 2012), and Platanus (v. 1.2.4) (Kajitani *et al.* 2014). CLC and SGA assembled over half of the expected genome size, though both assemblies were highly fragmented (Table 1). The Platanus assembly, which was intentionally focused on genic sequence, resulted in less than 1 Gb of contig sequence and was also extremely fragmented. BLAST+ searches indicated that 89%, 88%, and 76% of transcripts in the *B. variegata* reference transcriptome were at least partially included in the CLC, SGA, and Platanus genome assemblies, respectively (Supplementary Figure S3). Given the level of fragmentation, the CLC assembly was scaffolded against the *B. v. variegata* transcriptome with SCUBAT2 (G. Koutsovoulos, https://github.com/GDKO/SCUBAT2). SCUBAT2 assigned 73,298 CLC contigs to 13,300 paths (*i.e.*, sets of contigs linked by exons from a single transcript).

**Table 1:** Assembly comparison

	CLC	SGA	Platanus
Repeat-subtracted reads	No	Yes	Yes
Total contig length (Gb)	4.65	4.22	0.86
Number of contigs (×10^6^)	4.37	7.33	4.59
Contig N50 length (bp)	1815	823	229

### Reduced representation sequencing using nonrepetitive baits

Candidate sequences for bait design were chosen based on uniqueness, correct assembly, and minimal redundancy, as described in the Materials and Methods. Baits were synthesized for 3983 SCUBAT2 paths (including 2407 with inferred *B. bombina* orthologs), 35 CLC contigs matching genes of interest, and 982 CLC contigs without known gene association (total: 5000 targets and 20,000 baits, Supplementary File S4). The 4763 ARC-assembled loci in the *B. variegata* mapping reference had a mean length of 673 bp, more than twice the length of the 250 bp bait region. Addition of the complete CLC contigs for the remaining 237 loci resulted in a total sequence length of 4.5 Mb.

On average, each F0, F1, or F2 sample had 1,306,372 deduplicated, on-target read pairs. Only four samples had fewer than 500,000 such read pairs and belonged to one poorly performing enrichment pool. The average percentage of unique reads on target per readset was 19.8 (range: 9.5–27.1%, excluding samples from the poorly performing pool). The average number of post-QC read pairs per sample was 4,768,367. Mapping an unenriched readset of this size to the whole genome would equate to 0.17× coverage. The observed mean coverage of the 4.5 Mb mapping reference was 147×, representing about 865-fold enrichment. The read coverage across the 5000 targets appeared to be normally distributed (Supplementary Figure S4), but we noted a potential bias when mapping the *B. bombina* grandparent reads to the separate *B. variegata* and *B. bombina* references. The average ratio of reads mapped to the conspecific instead of the heterospecific reference was 1.1. However, this appeared to be the result of a small number of loci with large discrepancies (Supplementary Figure S5), as the median ratio was one.

### Diplotyping and linkage mapping

Diplotypes (BbHOM, HET, and BvHOM) were inferred for the two grandparents, the three F1 parents, and the 162 F2 offspring (see Supplementary File S5 for the full diplotype matrix). Diplotype inference failed for 136 targets, including 77 for which no variant positions were detected. Among the 4864 successfully clustered targets, only 25 had more than five missing diplotypes. Support estimates were greater than 10 ln likelihood units for 99.3% of the dataset (Supplementary Figure S6).

Of the 4864 targets, 4660 were grouped into 12 LGs by Lep-MAP3, matching the published haploid chromosome number (Morescalchi 1965; Manilo et al. 2006). We repeated the Lep-MAP3 analysis with the same dataset but replacing the data for 327 loci where the F0 grandparents and the F1 parents did not have the expected diplotype set of BvHOM, BbHOM, HET, HET, and HET. For these 327 loci, the rescored data using manually selected variants were used (see Materials and Methods). From this set, 154 were mapped in the first analysis. Based on manually selected variants, 138 of these 154 were again mapped and 16 were not. One locus moved from a terminal position of LG5 (180.86 cM) to the middle of LG4 (115.47 cM). The second analysis placed another locus at the exact same position (0 cM on LG6) but shifted all other loci in this LG by 3–4 cM, including another 11 rescored loci. Their displacement relative to neighboring loci ranged from 0 to 0.62 cM. Of the remaining loci in this set, 103 were placed at the exact same map position and only six moved by more than 2 cM (mean: 0.43 cM, maximum: 3.71 cM). Loci that changed position were about evenly split into those that joined a bin, moved between bins or moved to a new, unique position. The second analysis also added 94 loci to the map.

**Figure 4:**
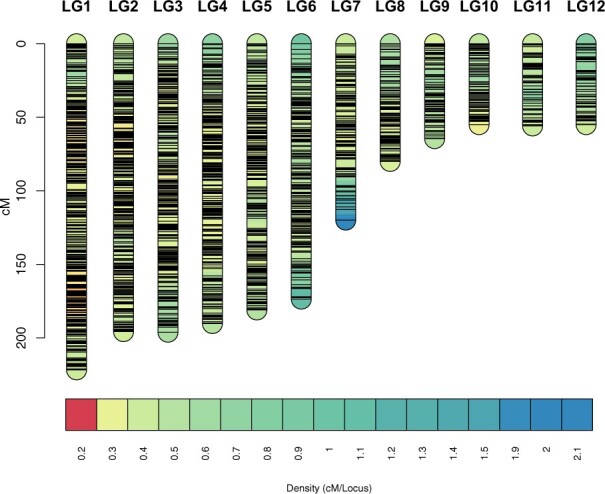
The *Bombina* linkage map. The linkage map was visualized with LinkageMapView (v. 2.1.2) (Ouellette *et al.* 2018). Horizontal bars represent marker loci. Colors indicate marker density in cM/locus from 0.2 (red) to 2.1 (blue). This figure is best viewed at maximum magnification.

The final map (Figure 4, Table 2) comprised 4755 loci and had a total length of 1588 cM with 2071 distinct map positions (bins), separated by 0.76 cM on average.

**Table 2:** Linkage map statistics

LG	# loci	# bins^*a*^	Max. gap [cM]^*b*^	Length [cM]	Length [cM] reduced
1	887	342	4.33	221.67	224.14
2	673	294	4.95	195.74	196.36
3	622	260	4.02	196.06	190.49
4	591	259	4.95	190.20	190.82
5	530	232	6.84	180.97	181.31
6	390	192	5.58	173.56	163.05
7	308	126	7.15	119.91	115.58
8	214	99	3.71	79.97	79.05
9	150	76	3.40	64.53	62.68
10	152	76	3.71	54.95	54.03
11	131	57	4.34	55.91	54.38
12	107	58	5.89	54.95	52.16
Total	4755	2071		1588.42	1564.05

All data are for the full map with 4755 loci, except for the last column which represents the reduced map of 2022 loci (one locus per cM position).

a Distinct map positions per LG.

b Maximum map interval between adjacent bins.

### Segregation distortion

**Figure 5:**
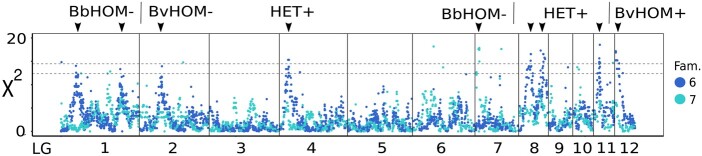
Segregation distortion, χ^2^, by family and linkage map position. Dashed horizontal lines are significance thresholds: the lower line is the Bonferroni correction based on the number of chromosome arms, and the upper line is the critical value for the Benjamini and Hochberg false discovery rate (the experiment-wise α is 0.05 for both). For each significant spike, which is indicated with an arrowhead, the genotype showing the strongest deviation is noted along with a (+) or (−) label, where (+) = excess and (−) = deficit. Groups of spikes with the same deviation are separated by vertical lines above the plot. For clarity, 22 observations from 20 loci with χ^2^ > 20 are excluded from the plot.

Across all LGs, there were nine distinct spikes in χ^2^ estimates that exceeded a lower significance threshold (the Bonferroni correction based on the number of chromosome arms), and six of these also exceeded an upper threshold (the critical value for the Benjamini and Hochberg false discovery rate; Figure 5). Eight spikes were only observed in family 6, but for some family 7 showed the same trend (LG1 right-hand spike, LG8 right-hand spike, and LG11). One spike on LG7 was restricted to family 7. Based on the diplotype with the strongest deviation, there were four spikes with a HET excess, three with a BbHOM deficit and one each with a deficit and an excess of BvHOM diplotypes. Overall, 144 and 75 mapped loci exceeded the lower and upper significance thresholds, respectively, in at least one family. Most of these were in spikes supported by many loci. We counted only 35 distinct outlier loci (0.7% of those mapped) with χ^2^ estimates larger than the lower (Bonferroni) threshold (see also Supplementary Figure S7). Supplementary File S6 lists the χ^2^ estimates for all mapped loci.

### Map validation

The reduced map with only one locus per map position was computed after all loci with greater than expected χ^2^ estimates had been removed (Supplementary File S1 and Figure S7) and one locus per bin had been chosen. That selection process was based on pairwise differences in inferred F2 diplotypes (*n *=* *162) among all loci within a bin. For 185 bins (17% of all bins with two or more loci), the maximum number of differences across locus pairs exceeded three (see Supplementary File S1 for details). In essentially all such cases, the removal of a single outlier locus reduced that maximum to three or less. A single locus was then picked at random from all bins. The map was recomputed for the resulting list of 2022 loci and consisted of 12 LGs with very similar lengths compared to the full map (Table 2). The cM positions of the 2022 markers were remarkably concordant on both maps (Supplementary Figure S8). Changes in marker order occurred in only five small groups of neighboring loci (max. displacement: 2.52 cM). There were also some differences in gap lengths and gap positions. The four most pronounced discrepancies are highlighted in Supplementary Figure S8.

Pairwise LOD scores and recombination fractions for each LG were computed for loci on the reduced map (Supplementary Figure S9). They provide strong statistical support for the inferred marker order.

### Large-scale synteny

**Figure 6:**
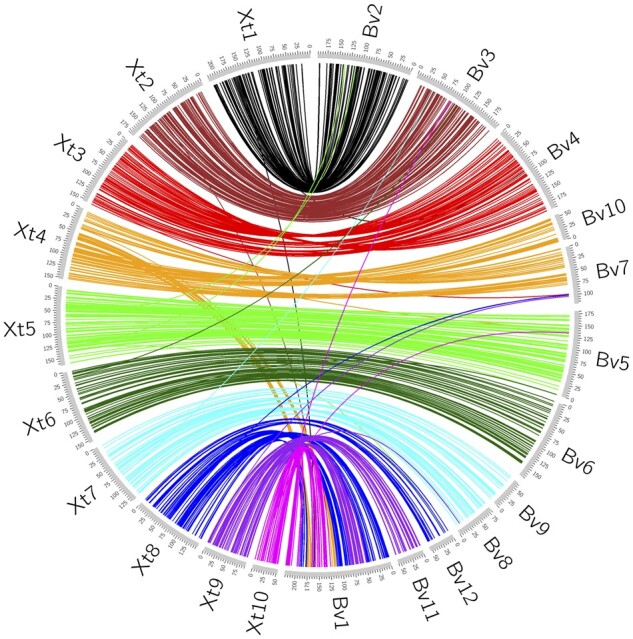
Synteny between *B. variegata and X. tropicalis*. Circos (v0.69-6) (Krzywinski *et al.* 2009) plot of 737 *B. variegata* target sequences from the 12 LGs (Bv, unit is cM) aligned against the *X. tropicalis* genome assembly (Xt, unit is Mb) with BLAST+ (v. 2.9.0) (Camacho *et al.* 2009).

We aligned the 5000 *B. variegata* target sequences against the *X. tropicalis* genome assembly (NCBI GCA_000004195.4 Bredeson *et al*., in prep) using BLAST+ (v. 2.9.0) (Camacho *et al.* 2009), with flags -task blastn -evalue 1E-10. Even with the large evolutionary divergence, 737 targets from the 12 LGs had hits to the *X. tropicalis* assembly, and the best blast hit was extracted. The *Bombina* LGs demonstrate obvious synteny to the *X. tropicalis* chromosomes (Figure 6). In particular, we found 1:1 correspondence between *X. tropicalis* chromosomes 1, 2, 3, 5, and 6 with LGs 2, 3, 4, 5, and 6, respectively. We also noted several distinct differences, such as intrachromosomal variation within these five conserved chromosomes and the split of *X. tropicalis* chromosome 7 into LGs 8 and 9.

LG1 consists of several interleaved synteny blocks shared with *X. tropicalis* chromosomes 4, 8, 9, and 10. Lep-MAP3 (v. 0.2) (Rastas 2017) split LG1 from other LGs that are syntenic with these four chromosomes (LGs 7, 10, 11, and 12) with a low LodLimit setting of 8. Note that LodLimit = 19 was required to split LGs 4 and 5 and to obtain the expected total of 12 LGs overall. There is thus no indication that LGs 1, 7, 10, 11, and 12 were unduly partitioned. The marker order on LG1 was also strongly supported by pairwise LOD scores (Supplementary Figure S9).

Six of the 17 loci with stray alignments in Figure 6 have multiple BLAST+ hits to the *X. tropicalis* genome, including hits on the expected chromosome. The placement of these loci likely reflects paralogy. Translocations or mapping errors may explain the remaining 11 cases. None of the 17 loci had exceptional χ^2^ estimates in the context of their map positions (Figure 5).

### Sex-determining region

**Figure 7:**
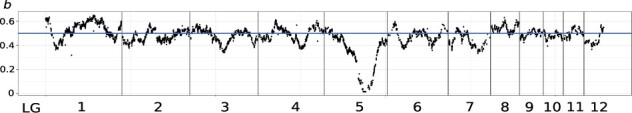
Estimated frequency of two sex-diplotype combinations among homozygous F2 individuals, *b*. See text for the definition of *b*. The global minimum on LG5 indicates the sex-determining region. The blue line represents the null hypothesis of *b *=* *0.5.

In an XY system or a ZW system, sex chromosomes would segregate in our crosses such that males cannot be BbHOM and females cannot be BvHOM in the SD region. Therefore, we can identify the SD region based on the frequency, *b*, of these two sex-diplotype combinations among homozygotes (see Materials and Methods). The global minimum across all LGs is on LG5 at 116.09 cM (*b *=* *0.0154), and the surrounding region (111–118 cM) on LG5 has a correspondingly low frequency (*b *<* *0.017; Figure 7). Based on the null hypothesis of *b *=* *0.5 for an autosomal locus, this region is statistically significant with *P *<* *10^−20^.

**Figure 8:**
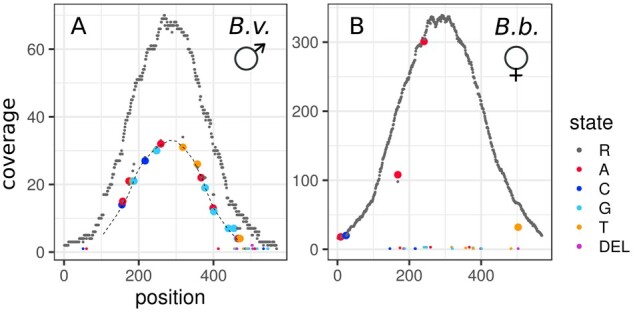
Raw read coverage at locus 5568 in the F0 generation. Plots follow the format of Figure 2: raw read coverage (*y*-axis) is shown for each reference position (*x*-axis) and sequence state (R = reference state) for F0 *B. variegata* (A) and F0 *B. bombina* (B). Nonreference states are highlighted in color. Variants in the sex-linked haplotype (A) are connected with a dashed line.

In order to identify the heterogametic sex, we searched the cluster plots for instances where males were strongly associated with particular clusters, estimated as *pMaleInMaleClusters* (see Materials and Methods). This statistic had a mode at 0.5 and a mean of 0.5534. Two *pMaleInMaleClusters* outliers were identified, and both loci are located near the identified SD region. For locus 5568 (LG5, 109.33 cM), *pMaleInMaleClusters was* 0.976, and for locus 4146 (LG5, 125.00 cM) it was 0.954. We identified a strongly diverged haplotype in the F0 *B. variegata* male at locus 5568 (Figure 8). This haplotype was inherited by the F1 father and by 59 of the 61 F2 offspring that were unambiguously male. Only 1 of the 60 high-certainty female F2 offspring carried this haplotype. These findings imply an XY system. Closer inspection of locus 4146 revealed that the *B. bombina* grandmother had a duplication of the target region on one chromosome and a deletion on the other. This indel configuration produced the extreme *pMaleInMaleClusters* estimate (Supplementary Figure S10). No outliers were observed in the analogous statistic, *pFemaleInFemaleClusters*. There is, therefore, no indication that *B. bombina* has a ZW system that could be competing with the *B. variegata* XY system in hybrids.

## Discussion

The new linkage map will be used as a tool to better understand the conundrum of abundant hybridization despite ancient divergence in the *Bombina* hybrid zone. Every step in its development was undertaken with this goal in mind. We produced genome assemblies, including one of the highly repetitive genome fraction, in order to identify nonrepetitive target sequences that could reliably report *B. bombina vs B. variegata* ancestry. The newly developed diplotyping approach efficiently extracts the ancestry signal while suppressing noise from overmerged reads that are an inevitable part of targeted enrichment (McCartney‐Melstad *et al.* 2016). Our strategy allowed 95% of the 5000 enrichment targets to be integrated into a linkage map. Its robustness was underscored by several validation steps, including the observed large-scale synteny with *X. tropicalis*. As a first biological insight, we located the *Bombina* SD region to 7 cM on LG5. The linkage map is our current best hypothesis about the order of markers on the 12 *Bombina* LGs, with a resolution of 0.03 cM. It will be refined through its application to deeper recombinant generations in the hybrid zone. To enable this next step and in keeping with our avoidance of arbitrary thresholds, we provide comprehensive statistics on all mapped markers, including outliers, in Supplementary File S6 and all existing annotation in Supplementary Files S7 and S8.

Anuran genomes are, in general, large (average size 4.7 Gb, Gregory 2021) and have extensive repeat content [over 70% in *Oophaga pumilio* (Rogers *et al.* 2018) and *Leptobrachium leishanense* (Li *et al.* 2019a)]. However, the repeat composition is highly variable among taxa. While DNA transposons make up the largest fraction of repeats in *X. tropicalis* (Hellsten *et al.* 2010) and *L. leishanense* (Li *et al.* 2019a), LTR retrotransposons feature prominently in *Nanorana parkeri* (Sun *et al.* 2015) and *O. pumilio* (Rogers *et al.* 2018). In *Rhinella marina* (Edwards *et al.* 2018) and *Leptobrachium* (*Vibrissaphora) ailaonica* (Li *et al.* 2019b), around 50% of the assembled repeats are unannotated. Our high coverage short-read dataset produced a highly fragmented and partial genome assembly for the *B. variegata* grandfather of our mapping crosses. Analysis of the *B. variegata* repeat content identified *DIRS* retrotransposons as the most common repeat (38% of annotated repeat content), followed by terminal inverted repeat DNA transposons (15%) and *Crypton* transposons (11%). *DIRS* and *Crypton* belong to a small subset of transposable elements that use tyrosine recombinase (YR) to integrate into the genome (Poulter and Goodwin 2005). They each account for less than 2% of the repeat content in other anuran assemblies. Knowledge of the *Bombina* repeat content steered our bait design away from undesirable sequences without Repbase annotation, including the repeat with the highest copy number overall.

Because target capture was not perfect, off-target reads commonly aligned to and accumulated at one or both ends of the reference sequences. These reads introduced heterozygous variants that contradicted the variants elsewhere in the reference. This was expected for a highly repetitive genome, and our delayed-calling analysis pipeline was designed accordingly. Overmerging adds noise to the inheritance signal at a locus, reducing the power to call an individual’s genotype. However, late-calling eschews this low power early calling step: haplotypes were instead called from the combined read data of all individuals in a homozygous cluster (∼40) and thus at >1000-fold coverage (see Materials and Methods). When *N* is this large, the algorithm reports on the locus that generates the strongest inheritance signal, nothwithstanding any overmerging signal from other loci. Given that baits were designed from the *B. variegata* genome assembly, we also expected enrichment bias in heterozygous individuals. With delayed-called haplotypes, we allowed for such bias by maximizing the likelihood of an individual’s data over the admixture coefficient between haplotype pairs, coestimating bias. Diplotype calls are thus late, powerful, and robust to both overmerging and enrichment bias. Recent ancestry inference tools for whole-genome resequencing data have been similarly based on raw read counts rather than called genotypes (Corbett-Detig and Nielsen 2017; Schumer *et al.* 2020).

While the delayed calling stage of our analyses follows standard likelihood approaches, it relies on an initial automated clustering of individual’s raw data. To assess the properties of this clustering heuristic, we rescored a subset of 327 (6.5%) of loci by direct inspection [*i.e*., those that did not show the expected (BvHOM, BbHOM, HET, HET, and HET) diplotype estimates in the F0 and F1 generations; see Materials and Methods]. Although such deviations are not necessarily problematic, this subset included some challenging loci. Structural variation was common, mainly homozygous or heterozygous whole-locus deletions, most of which could not be mapped. A number of loci had strongly distorted segregations and remained unmapped after rescoring. Among the loci that were added to the map (*n *=* *95), there were 70 for which more than three diplotype clusters had been inferred, reflecting distinct haplotypes (alleles and/or overmergings) within one or both of the grandparents. These 70 represent about 25% of such loci on the map. While the analysis pipeline is set up to extract haplotypes from more than two clusters and compares all candidate pairs within the likelihood framework, within-taxon sequence variation appears to be the most difficult case for the clustering heuristic. This is not surprising, given its design for between-taxon variation. Nonetheless, at locus 5568, the heuristic produced the same partition of the data as direct inspection, despite the strongly diverged *B. variegata* haplotype (Figure 8 and Supplementary Figure S11). Moreover, the rescoring of loci that were part of the original map brought little change: 95% of these loci were displaced from their nearest neighbors by less than 2 cM.

As expected (Fishman and McIntosh 2019), there were two types of loci with significant segregation distortion: those located in distinct χ^2^ spikes, formed by a large number of closely neighboring loci, and those that were true outliers in their local map context (Figure 5). The fact that independently scored loci within each spike produced a gradual change in χ^2^ leading to a local maximum suggests a biological cause. While overmerging produces single loci with heterozygote excess, it is not a plausible explanation for the four observed HET+ spikes. One would have to assume that overmerging increases and decreases gradually along the map and can differ between families. Instead, the χ^2^ spikes likely reflect hybrid incompatibilities or, especially in cases of homozygote deficit in one taxon, inbreeding depression in the full-sib F1 crosses (Fishman and McIntosh 2019). The family differences indicate genetic variation within one or both grandparents that affects F2 fitness. There were no significant genotype associations between pairs of loci from different χ^2^ spikes in family 6 (analyses not shown). The small number of true outlier loci above the Bonferroni threshold (*n *=* *35) underscores the reliability of our diplotyping.

While we expected low chromosome-level variation between *X. tropicalis* and *Bombina*, our visualization of these results is remarkably stark. The large-scale synteny as well as the presence of only a few stray alignments, all of which appear to be single, isolated hits, suggests that the overall structure of the linkage map agrees with the *X. tropicalis* chromosome structure and substantiates the linkage map construction. If we assume the *Bombina* map estimation is error free for the five LGs (2, 3, 4, 5, and 6) that show 1:1 synteny with five *X. tropicalis* chromosomes and that errors are Poisson distributed in the intervals between the 737 aligned markers, evenly distributed over 12 chromosomes, then the map error rate estimate is 0.015. This estimate is conservative, because the five “error free” chromosomes have more markers than assumed.

These observations also suggest that these five chromosomes were present in the Bombinanura ancestor and that the distinct chromosome boundaries have been maintained for the past ∼200 million years (Feng *et al.* 2017). The observed differences are similarly informative, suggestive of either biological diversity or linkage map construction error. Future exploration of these synteny patterns, particularly in comparison against additional chromosome-scale frog assemblies (Mudd 2019), will increase our understanding of anuran chromosome evolution.

We located the *Bombina* SD region on LG5 (111–118 cM) using the association between homozygote genotypes and sex in F2 offspring and, at nearby locus 5568 (LG5, 109.61 cM), we identified a haplotype in the F0 *B. variegata* male that is strongly associated with male sex in the F2 generation, indicating an XY system. Because this locus lies outside any nonrecombining SD region, the observed sex-linkage of the distinctive haplotype (Figure 8) in our crosses is fortuitous. In a preliminary analysis of wild-caught *B. bombina* and *B. variegata* samples from Romania, Poland, and the Czech Republic (*n *=* *35 per taxon), this haplotype occurred at a frequency of 0.13 in *B. variegata* and in both males and females. It happens to be present in the male grandparent and in phase with the Y haplotype. Male heterogamety was also established for *Bombina orientalis* (Kawamura and Nishioka 1977), the nearest relative of *B. bombina* and *B. variegata* (MRCA 4.6 mya, Nürnberger *et al.* 2016).

Similar to the situation in fish (Volff *et al.* 2007; Gammerdinger and Kocher, 2018 ), the identity of the sex chromosome in amphibians can vary between closely related species and even among populations within a species (Miura 2017; Jeffries *et al.* 2018). Nonetheless, not all chromosomes are equally likely to take on the SD role. In anuran XY systems, chromosome 1 (numbering by homology with *X. tropicalis*) features disproportionately across diverse genera, such as *Rana, Hyla*, and *Bufo* (Brelsford *et al.* 2013; Tamschick *et al.* 2014; Miura 2017; Jeffries et al. 2018). All other known XY cases involve chromosomes 2, 3, and 5, and in the genus *Rana*, switches to chromosome 5 occur more often than expected by chance (Jeffries *et al.* 2018). Also, known genes of the SD pathway are located on chromosome 1 (*Dmrt1* and *Amh*) and 5 (*FoxL2*, Jeffries *et al.* 2018). The observed pattern could arise if a relatively small number of genes in the vertebrate sex determination cascade alternated in assuming the master SD role (Volff *et al.* 2007; Graves and Peichel 2010; Herpin and Schartl 2015; Furman and Evans 2016). The *Bombina* sex chromosome is indeed homologous to *X. tropicalis* chromosome 5, but the *FoxL2* ortholog marker is located at 39.83 cM, well outside the SD region. Thus, the genic content in the *Bombina* SD region is presently unknown.

Our ability to delineate the SD region relied on the heterogametic recombination rate. In fact, the gradual decline of *b* toward its global minimum on LG5 (Figure 7) was caused entirely by recombination in the F1 male (Supplementary Figure S2). Chiasma counts in *B. variegata* (Morescalchi 1965; Morescalchi and Galgano 1973) suggest that the female:male crossover rate is around 1.3 and that recombination in either sex is not localized to particular chromosome regions. These observations contrast with the findings in other anurans, such as *Rana, Hyla*, and *Xenopus* (Brelsford *et al.* 2016a, 2016b; Furman and Evans 2018), where the female recombination rate exceeds that in males up to fourfold (in one case even 75-fold, Rodrigues *et al.* 2013) and male crossovers are largely restricted to chromosome ends. The “recombination landscape” observed in these three genera is typical for vertebrates in general (Sardell and Kirkpatrick 2020). It should favor XY sex chromosome turnover (Jeffries *et al.* 2018; Sardell and Kirkpatrick 2020) and contribute to the typically greater differentiation near chromosome centers relative to the ends between closely related species (Haenel et al. 2018; Sardell and Kirkpatrick 2020). We expect that these dynamics play a lesser role in *Bombina*.

The age of the *Bombina* SD system could be inferred from a phylogenetic analysis of sex linkage across sister taxa. Alternatively, X-Y sequence divergence could be estimated from loci in the nonrecombining region (Charlesworth *et al.* 2005). However, none of the loci in the 7 cM interval, where *b* is at or near its minimum, had sex-linked haplotypes, and they therefore presumably bracket the SD region. Conceivably, the X and Y sequences closely associated with the SD locus are so diverged that baits derived from one haplotype do not capture the other. Such a locus may thus not be mapped. However, none of the 5000 loci showed the particular segregation patterns expected in this case (Supplementary Figure S12). We, therefore, suspect that the SD region is not large. A small nonrecombining region would be consistent with a young SD system but not proof (Charlesworth 2019), because some old SD systems provide counterexamples (*e.g.*, Vicoso *et al.* 2013).

While whole-genome sequence represents the ultimate genomic resource, it is rarely attainable and commonly nonessential. For many evolutionary questions, it is sufficient to sample populations for small portions of genomes placed on a linkage map. This is particularly true for genome-wide hybrid zone studies, where linkage disequilibria require analysis in a map context, but increased SNP detection provides no additional information after all segregating ancestry tracts have been marked. This applies irrespective of genome size. The approach is therefore particularly attractive for hybridizing species with large genomes, provided that markers from the nonrepetitive part of the genome can be identified and reliably scored. The new *Bombina* linkage map fulfills these criteria. Knowledge of the SD region and of the large-scale synteny with *X. tropicalis* broadens our scope for inference. In short, the map provides the much-needed tool to take the analysis of this classic study system to a new level.

## Data availability

The following files have been submitted to Figshare: the photographs of histological sections (Supplementary Figure S1), the plots of pairwise recombination rates and LOD scores (Supplementary Figure S9), the Mathematica script for the diplotype analysis (Supplementary File S2), data resources needed to repeat the analysis, annotations and the final linkage map file (Supplementary File S6). Supplementary File S9 is a guide to the resources in the archive. Supplementary Files S10 and S11 provide the REPdenovo assembly and the *B. variegata* mapping reference, respectively. The scripts for rescoring a subset of loci based on manually selected variants have been submitted to GitHub (https://github.com/beanurn/diplocheck). WGS reads, the CLC assembly and the read sets from enriched libraries have been deposited in the European Nucleotide Archive (ENA) at EMBL-EBI under accession number PRJEB35099 (https://www.ebi.ac.uk/ena/browser/view/PRJEB35099). Supplementary material is available at figshare: https://doi.org/10.25387/g3.14216987 and at G3 online.
